# A Pilot Study to Assess Effects of Long-Term Inhalation of Airborne Particulate Matter on Early Alzheimer-Like Changes in the Mouse Brain

**DOI:** 10.1371/journal.pone.0127102

**Published:** 2015-05-20

**Authors:** Dhaval P. Bhatt, Kendra L. Puig, Matthew W. Gorr, Loren E. Wold, Colin K. Combs

**Affiliations:** 1 Department of Basic Sciences, UND School of Medicine and Health Sciences, Grand Forks, North Dakota, United States of America; 2 Duke Molecular Physiology Institute, Duke University, Durham, North Carolina, United States of America; 3 Dorothy M. Davis Heart and Lung Research Institute and Department of Physiology and Cell Biology, The Ohio State University College of Medicine, Columbus, Ohio, United States of America; 4 College of Nursing, The Ohio State University, Columbus, Ohio, United States of America; Indiana School of Medicine, UNITED STATES

## Abstract

Exposure to air pollutants, including particulate matter, results in activation of the brain inflammatory response and Alzheimer disease (AD)-like pathology in dogs and humans. However, the length of time required for inhalation of ambient particulate matter to influence brain inflammation and AD pathology is less clear. Here, we studied the effect of 3 and 9 months of air particulate matter (<2.5 μm diameter, PM_2.5_) exposure on brain inflammatory phenotype and pathological hallmarks of AD in C57BL/6 mice. Using western blot, ELISA, and cytokine array analysis we quantified brain APP, beta-site APP cleaving enzyme (BACE), oligomeric protein, total Aβ 1–40 and Aβ 1–42 levels, inducible nitric oxide synthase (iNOS), nitrotyrosine-modified proteins, HNE-Michael adducts, vascular cell adhesion molecule 1 (VCAM-1), glial markers (GFAP, Iba-1), pre- and post- synaptic markers (synaptophysin and PSD-95), cyclooxygenase (COX-1, COX-2) levels, and the cytokine profile in PM_2.5_ exposed and filtered air control mice. Only 9 month PM_2.5_ exposure increased BACE protein levels, APP processing, and Aβ 1–40 levels. This correlated with a concomitant increase in COX-1 and COX-2 protein levels and a modest alteration in the cytokine profile. These data support the hypothesis that prolonged exposure to airborne particulate matter has the potential to alter brain inflammatory phenotype and promote development of early AD-like pathology.

## Introduction

Alzheimer’s disease (AD) is a progressive dementia characterized by altered processing of amyloid precursor protein (APP), formation of beta-amyloid plaques (Aβ), hyper-phosphorylated tau containing neurofibrillary tangles, and synaptic loss in the brain. In 2010, there were 5.3 million Americans with AD [1] and this number is expected to rise to 13.8 million by 2050 [2]. During 1979–2010, while the total mortality in the U.S. decreased, the total number of deaths resulting from AD increased by 68% during 2000–2010 [3]. From all the known risk factors, advancing age is the greatest risk factor for AD. However, this inconsistent rise in AD-related deaths cannot be explained by an overall increase in the aging population. Therefore, there is a need to seek putative risk factors that can not only provide measurable association with disease pathology but can also be modulated at the population level.

Central nervous system inflammation is a well-accepted feature of AD that is hypothesized to contribute to the disease pathology and its progressive nature [4]. Besides age and other genetic risk factors, environmental factors like air pollution can influence peripheral and brain inflammation. In addition to poisonous gases, organic compounds and metals, polluted air contains particulate matter that ranges from coarse (diameter between 2.5–10 μm, PM_10_) to fine particles (diameter <2.5 μm, PM_2.5_) and ultrafine particles (diameter <0.1 μm, PM_0.1_). As per the National Ambient Air Quality Standards (NAAQS), annual exposure to PM_2.5_ must be below 15 μg/m^3^ of ambient air [5]. Combustion and industrial activities result in PM_2.5_ formation mainly comprised of organic and inorganic compounds such as sulfates, nitrates, carbon, ammonium, hydrogen ions, lipopolysaccharides, metals and water [6]. This diverse set of particulate matter constituents has traditionally been associated with pulmonary and cardiovascular diseases [6–8]. More recently, observational studies in humans living in polluted areas [9] and acute exposure studies in dogs with highly concentrated particulate matter and other air pollutants [10] have revealed that air pollution can influence the brain inflammatory phenotype and promote development of AD-like pathology. However, it is unclear whether PM_2.5_ exposure levels that meet NAAQS levels can influence AD progression.

Therefore, in this pilot study we used a long-term airborne particulate matter inhalation model in mice to mimic exposure to PM_2.5_ levels near the NAAQS and quantified changes in the brain inflammatory state and AD pathology. Based upon prior work using this paradigm to model PM_2.5_ exposure-dependent changes in the cardiovascular system we hypothesized that similar exposure times would be reasonable for examining the brain. Specifically, PM2.5 exposure for 3 months induced an early cardiovascular phenotype in prior work [11] and a more developed one at 9 months exposure [8]. We found that 9 months of exposure to PM_2.5_ increased Aβ levels and decreased full length APP protein levels correlating with an increase in protein levels of the APP proteolytic enzyme, BACE. These changes correlated with increases in COX-1, COX-2, and PSD-95 protein levels and a modest increase in the chemotactic cytokine profile. These data support the hypothesis that specific types or lengths of exposure to ambient airborne particulate matter even meeting NAAQS levels are capable of altering the brain inflammatory milieu and promoting progression of AD.

## Methods and Methods

### Reagents

Primary antibodies (Ab) against actin (sc-1616), α-tubulin (sc-8304), COX-2 (sc-1745), tau (sc-1995), and VCAM-1 (sc-8035) were purchased from Santa Cruz Biotechnology Inc. (Dallas, TX). Antibodies against BACE (5606), GFAP (3670), and PSD-95 (3450 XP) were obtained from Cell Signaling Technology Inc. (Danvers, MA). HNE-adducts Ab (393207) and synaptophysin Ab (MAB5258) were from EMD Millipore (Billerica MA). Antibodies for inducible-NOS (ALX-210-504-R100) and nitrotyrosine (ALX-804-204-C050) were from Enzo Life Sciences Inc. (Farmingdale, NY). The amyloid precursor protein Ab (Ab32136) was from Abcam (Cambridge, MA), COX-1Ab (160110) was from Cayman Chemical Company (Ann Arbor, MI), Iba-1 Ab (019–19741) from Wako Chemicals USA Inc. (Richmond, VA), A11 was a gift from Dr. Rakez Kayed and anti-phospho tau Ser 396/404 (PHF-1) was a gift from Dr. Peter Davies. Antibodies against selectively rodent Aβ (SIG-39151) and rodent or human Aβ (4G8, SIG-39200) were purchased from Covance (Princeton, NJ). All horseradish peroxidase conjugated secondary antibodies donkey anti-rabbit IgG (sc-2317), bovine anti-goat IgG (sc-2350), goat anti-mouse IgM (sc-2064), bovine anti- mouse IgG (sc-2371) were from Santa Cruz Biotechnology Inc. (Dallas, TX).

### Airborne particulate matter exposure

All animal use was approved by the Ohio State University Institutional Animal Care and Use Committee (#2013A00000074). Mice were provided food and water *ad libitum* and housed in a 12 h light:dark cycle. The investigation conformed to the National Research Council of the National Academies Guide for the Care and Use of Laboratory Animals (8th edition). Male C57BL/6 mice were exposed to concentrated air particulate matter (<2.5 μm diameter, PM_2.5_) using the Ohio air pollution exposure system for interrogation of systemic effects (OASIS-1), modified for long-term exposures, as previously described [8]. Briefly, 8 weeks old mice were exposed 6 h/day, 5 days/week for 3 or 9 months. Using this exposure paradigm, at 3 months an early cardiovascular phenotype was previously observed [11] and a more developed one at 9 months exposure [8]. A 7.7-fold concentration (65.7 ± 34.2 μg/m^3^) of PM_2.5_ was achieved in the exposure chamber compared to the ambient mean daily PM_2.5_ concentration (8.5 ± 3.7 μg/m^3^) at the study site in Columbus, OH. Control (filtered air, FA) mice received PM_2.5_ treatment that was filtered through a high-efficiency particulate air filter (Pall Life Sciences, East Hills, NY). The average yearly PM_2.5_ concentration normalized to the total exposure period (including the non-treatment days) was 11.7 ± 6.1 μg/m^3^, less than the standard annual average PM_2.5_ of 15 μg/m^3^ established by the NAAQS. The composition of the PM_2.5_ that the mice received during this study was dependent upon the air in Columbus during the time of exposure. This has been previously examined using x-ray fluorescence spectroscopy and characterized [12], where they were found to contain notably high levels of zinc and iron.

### Tissue preparation

Brains were dissected on ice and left hemispheres were flash frozen in liquid nitrogen for immunohistochemical analysis while the right was flash frozen in liquid nitrogen then stored at -80°C for cytokine array, immunoblotting, and Aβ ELISA. Based upon the importance of the temporal cortex as a region of AD-associated pathology, we elected to collect right temporal cortex regions for homogenizing in the cytokine array analysis buffer (RayBiotech, Norcross, GA). A portion of the supernatant was used for cytokine arrays. The remaining supernatant and the pellet were again homogenized but now in radioimmunoprecipitation assay buffer (RIPA, 20mM Tris, pH 7.4, 150mM NaCl, 1mM Na_3_VO_4_, 10mM NaF, 1mM EDTA, 1mM EGTA, 0.2mM phenylmethylsulfonyl fluoride, 1% Triton, 0.1% SDS, and 0.5% deoxycholate) with protease inhibitors (AEBSF 1mM, Aprotinin 0.8μM, Leupeptin 21μM, Bestatin 36μM, Pepstatin A 15μM, E-64 14μM). The supernatant was collected, protein concentration determined by Bradford protein assay [13], and stored at -80°C for immunoblotting and Aβ ELISA. Parietal cortices were isolated for DNA extraction using a Genejet Genomic DNA isolation kit (Thermo Fisher Scientific Grand Island NY), for 5-methylcytosine ELISA.

### Immunoblotting

Equal amounts of protein (10–20 μg/well) were loaded and separated using SDS-PAGE (7–15%). Proteins were transferred to PVDF membranes and blocked with 5% BSA in Tris-buffered saline containing 0.05% Tween (TBS-T). Blots were incubated overnight with primary antibodies (1:1000) at 4° C, washed in TBS-T and incubated with secondary antibodies (1:10,000) at room temperature for 1 h. Protein bands were visualized with a Amersham ECL substrate (GE Healthcare Bio-Sciences, PA) using a UVP Bioimaging System (Upland, CA). Image capturing and analysis was performed with Vision Works imaging software (Upland, CA). Optical densities obtained for all proteins were normalized to their respective α-tubulin or actin loading controls and the data is expressed as percentage over control levels.

### Immunohistochemistry

Left brain hemispheres was embedded in the optimal cutting temperature (OCT) compound and serial 40 μm sections were cut using a cryostat (Leica Microsystems Inc, IL). Endogenous peroxidase activity was suppressed by incubating sections, mounted on gelatin-subbed slides, with 0.3% hydrogen peroxide and were blocked with phosphate buffered saline containing 0.5% BSA, 0.05% goat serum 0.001% Triton-X, and 0.02% azide (PBS solution) for 1 h at room temperature. Primary antibodies (1:250–1:1000) in PBS solution were incubated with sections at 4°C overnight, washed in PBS solution and incubated with the biotinylated secondary antibodies (1:2000–1:5000) followed by incubation with avidin/biotin. Stains were visualized using a peroxidase-based Vector VIP chromogen (Vector Laboratories, CA). Images were taken using an upright Leica DM1000 microscope and Leica DF320 digital camera system.

### Aβ Enzyme-Linked Immusorbent Assay (ELISA)

Mouse Aβ 1–40 and 1–42 ELISA kits were obtained from Life Technologies (Grand Island NY) and 150 μg temporal cortex lysates were analyzed in duplicates as per the manufacturer’s protocol.

### 5-methylcytosine ELISA

DNA isolated from parietal cortices of filtered air and particulate matter exposed mice was used to quantify relative levels of 5-methylcytosine DNA according to the manufacturer’s protocol (Zymo Research, Irvine, CA) using 100ng/brain.

### Dot blot

Approximately 100 ng of temporal cortex lysates (in not more than 5 μL total volume) for each sample was spotted onto PVDF membranes using a dot blot assembly. The membranes were blocked in 5% BSA and then processed as described above for western blot analysis.

### Cytokine array

A total of 1 mg protein of temporal cortex lysates was loaded onto the antibody-coated inflammatory cytokine array (RayBiotech Inc, Norcross, GA) nitrocellulose membranes. The membranes were incubated with secondary antibodies and the cytokines were visualized as per manufacturer’s protocol.

### Statistical analysis

Data represent means ± SD, n = 4 for 9 month filtered air controls and n = 5 for 9 month particulate matter mice and n = 5 for 3 month filtered air controls and n = 5 for 3 month particulate matter mice. Statistical analysis was performed using a t-test and the significance level was set at P <0.05 (*).

## Results

### Exposure to airborne particulate matter increased Aβ levels but did not affect tau hyper-phosphorylation

**Fig 1:**
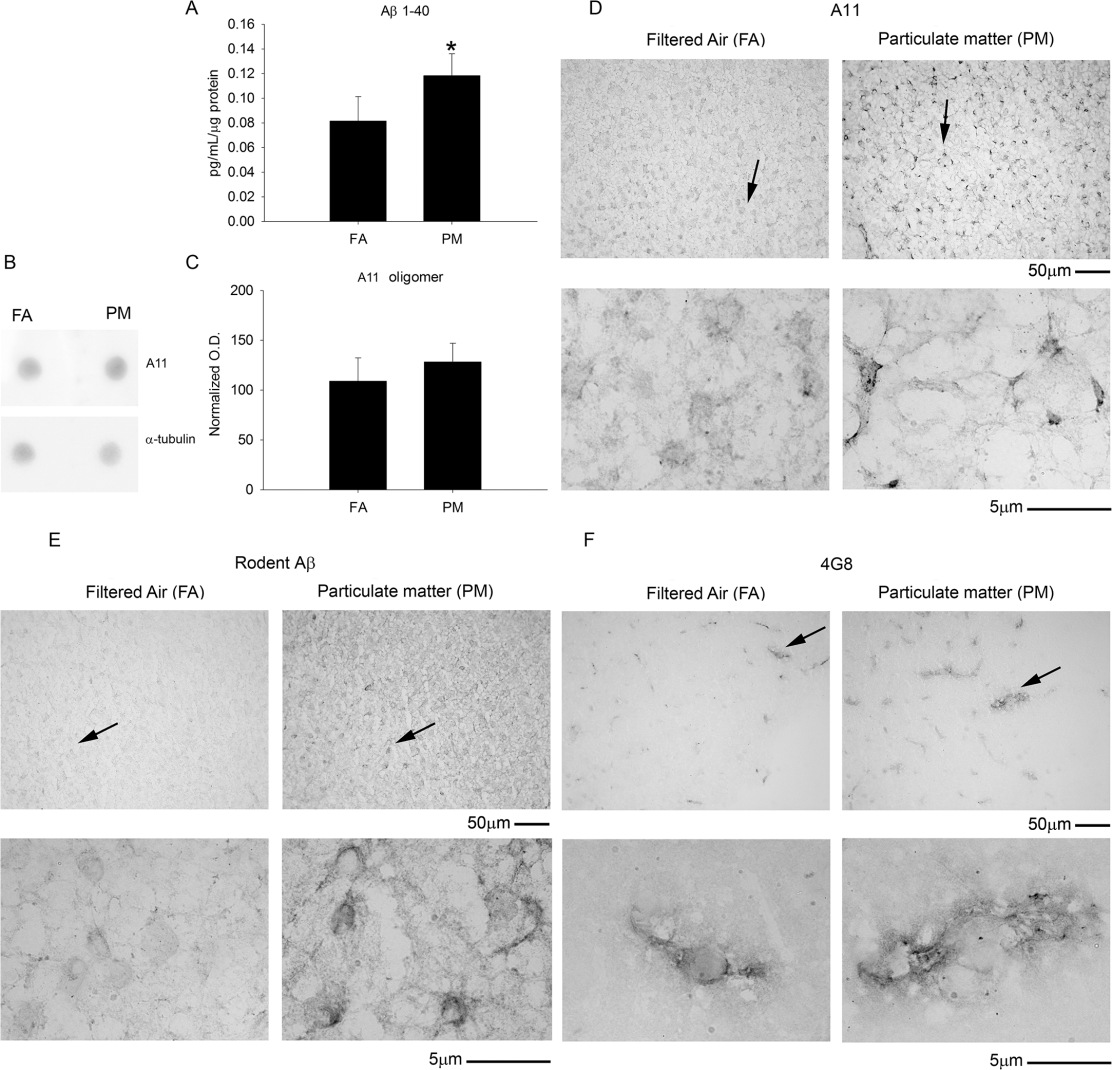
Brain amyloid beta 1–40 (Aβ 1–40) levels and Aβ immunoreactivity increased with 9 month airborne particulate matter exposure while no change in oligomerized protein staining was observed. Brains of 9 month exposed filtered air (FA) or PM_2.5_ (PM) mice were collected with the left hemisphere fixed for immunohistochemistry and the right temporal cortices collected for biochemical analysis. *A*. Rodent Aβ 1–40 levels from FA and PM temporal cortices were quantified by ELISA, *p<0.05. *B*. Temporal cortex lysates from FA and PM brains were used for dot blot analysis using A11 anti-oligomer antibody and α-tubulin (loading control). *C*. A11 optical densities were normalized to their respective loading control, averaged (+/-SD), and graphed. Brains were immunostained using *D*, anti-A11 antibody to visualize oligomeric protein, *E*, anti-rodent specific Aβ antibody, and *F*, anti-Aβ antibody 4G8. Images shown are representative from the temporal cortex. Arrows indicate regions taken for higher magnification.

**Fig 2:**
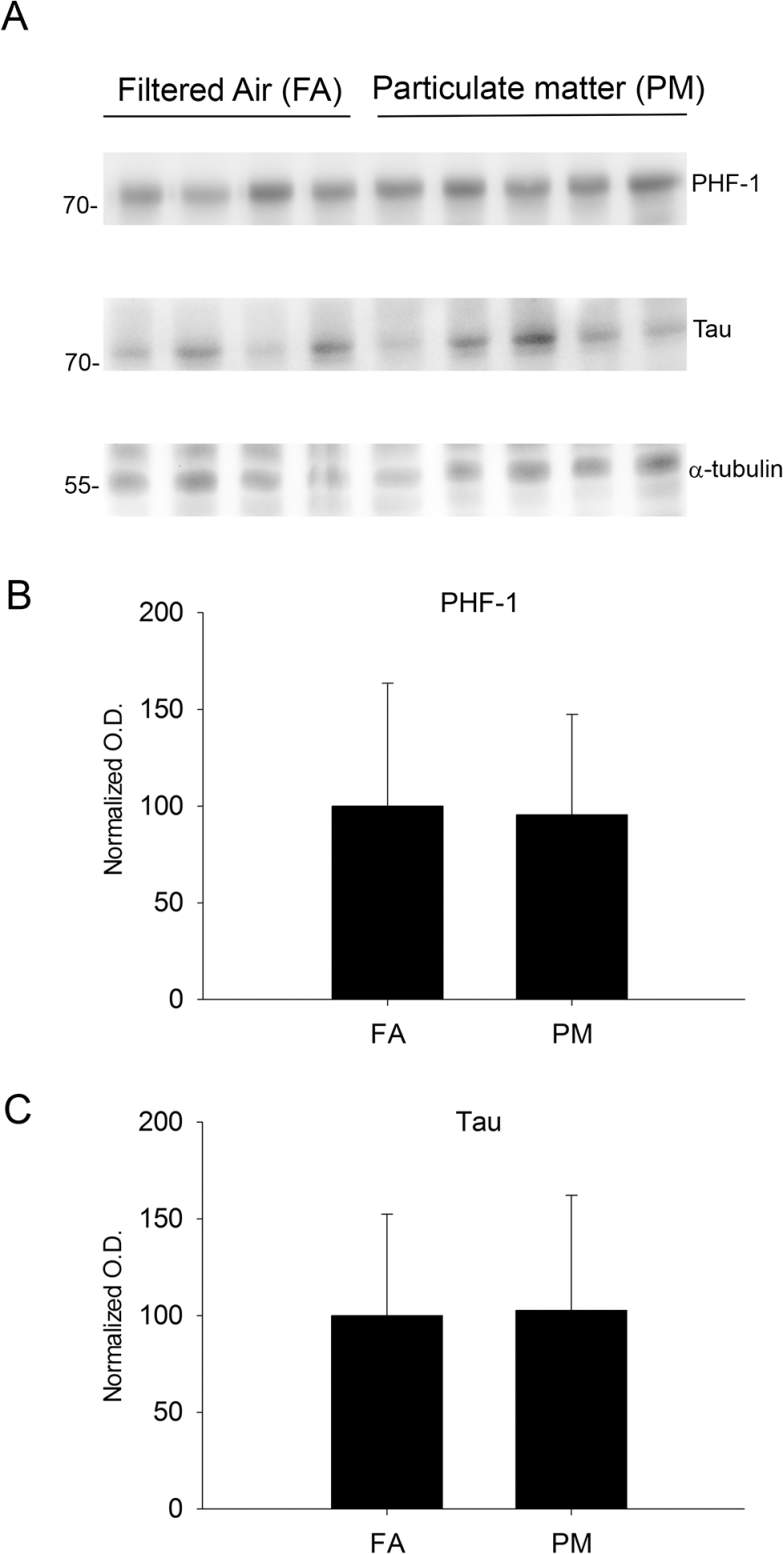
Brain microtubule-associated protein (tau) and its phosphorylated form (PHF-1) were not altered by 9 month exposure to airborne particulate matter. Brains of 9 month exposed filtered air (FA) or PM_2.5_ (PM) mice were collected with the left hemisphere fixed for immunohistochemistry and the right temporal cortices collected for biochemical analysis. *A*. Temporal cortex lysates from FA and PM brains were used for western blot analysis using anti-phopsho tau (PHF-1), anti-tau (loading control for PHF-1), and anti-α-tubulin (loading control for tau), A11 anti-oligomer antibody and α-tubulin (loading control). *B*. PHF-1 optical densities were normalized to their tau loading controls, averaged (+/-SD), and graphed. *C*. Tau optical densities were normalized to their α-tubulin loading controls, averaged (+/-SD), and graphed.

Since Aβ plaques and hyper-phosphorylated tau are two well characterized hallmarks of Alzheimer’s disease, using ELISA and western blot analysis we quantified their levels in mouse cerebral temporal cortex, respectively. The Aβ 1–40 levels, as measured by ELISA, were significantly higher in airborne particulate matter (diameter <2.5 μm, PM_2.5_) compared to the filtered air controls (Fig 1) in the 9 month exposure group. Consistent with this data, immunochemical staining also demonstrated robust Aβ immunoreactivity in the temporal cortex of the PM_2.5_ exposed mice using both a rodent specific anti-Aβ antibody and the anti-Aβ antibody, 4G8 (Fig 1). The Aβ 1–42 levels were also quantified but were below the detection limit for the assay (data not shown). On the other hand, no changes in tau protein or its hyper-phosphorylated form (PHF-1) were observed using western blot analysis (Fig 2). To determine if elevated Aβ 1–40 levels correlated with increased oligomeric proteins levels, we performed dot blot analysis and immunostaining using an antibody specific for oligomeric peptide, (A11). There was no significant change observed in total oligomeric protein from dot-blot analysis (Fig 1). This was consistent with no robust difference in A11 staining in the temporal cortex (Fig 1). No significant changes in Aβ or tau phosphorylation were observed in the 3 month PM_2.5_ group compared to FA controls (data not shown). These data suggest that 9 months of exposure to airborne particulate matter induced modest increases in Aβ accumulation.

**Fig 3:**
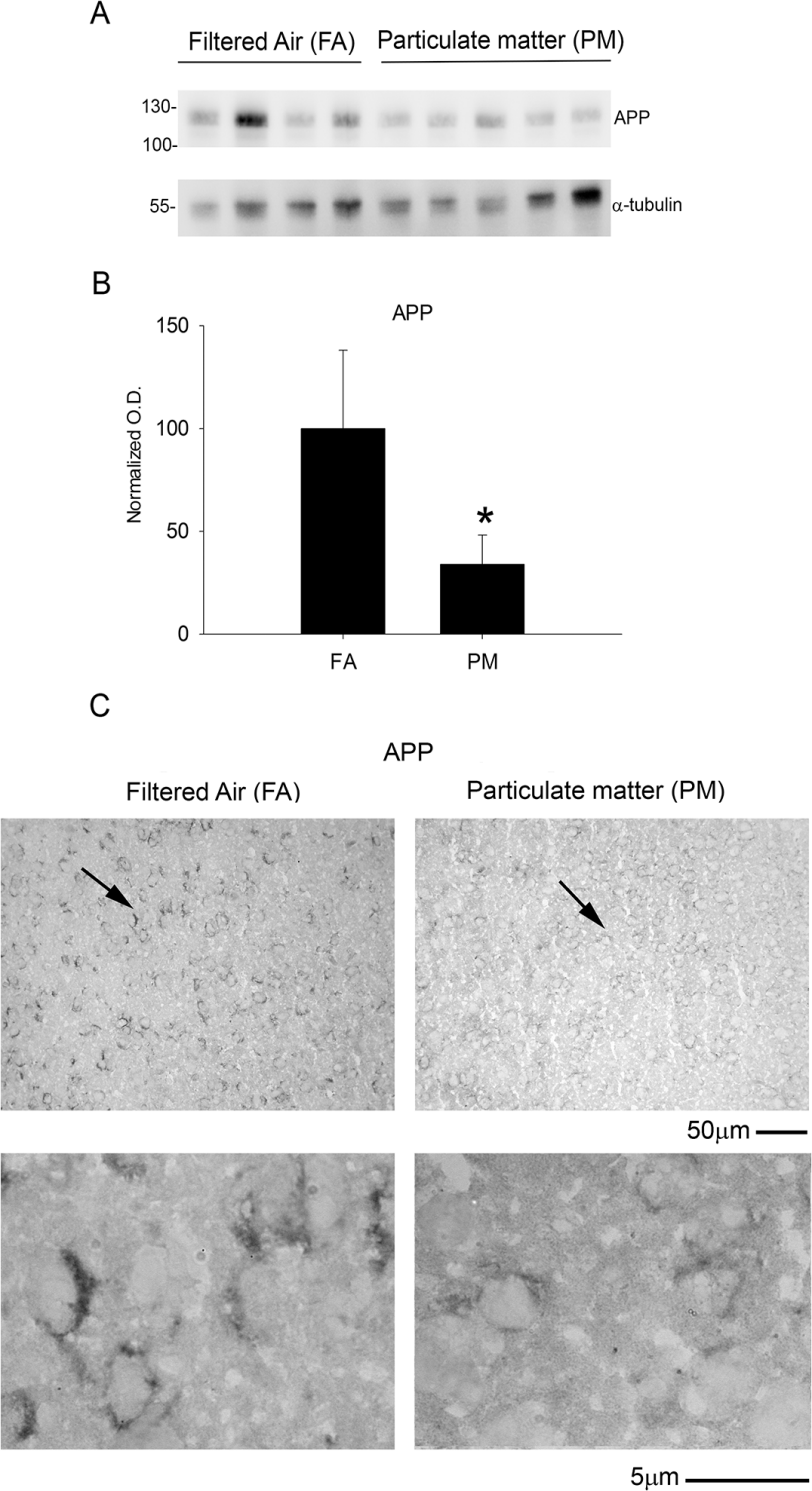
Brain amyloid precursor protein (APP) levels were reduced after 9 month exposure to airborne particulate matter. Brains of 9 month exposed filtered air (FA) or PM_2.5_ (PM) mice were collected with the left hemisphere fixed for immunohistochemistry and the right temporal cortices collected for biochemical analysis. *A*. Temporal cortex lysates from FA and PM brains were used for western blot analysis using anti-APP, Y188 antibody and α-tubulin (loading control). *B*. APP optical densities were normalized to their respective loading controls, averaged (+/-SD), and graphed, *p<0.05. *C*. Brains were immunostained using anti-APP, Y188 antibody. Images shown are representative from the temporal cortex. Arrows indicate regions taken for higher magnification.

**Fig 4:**
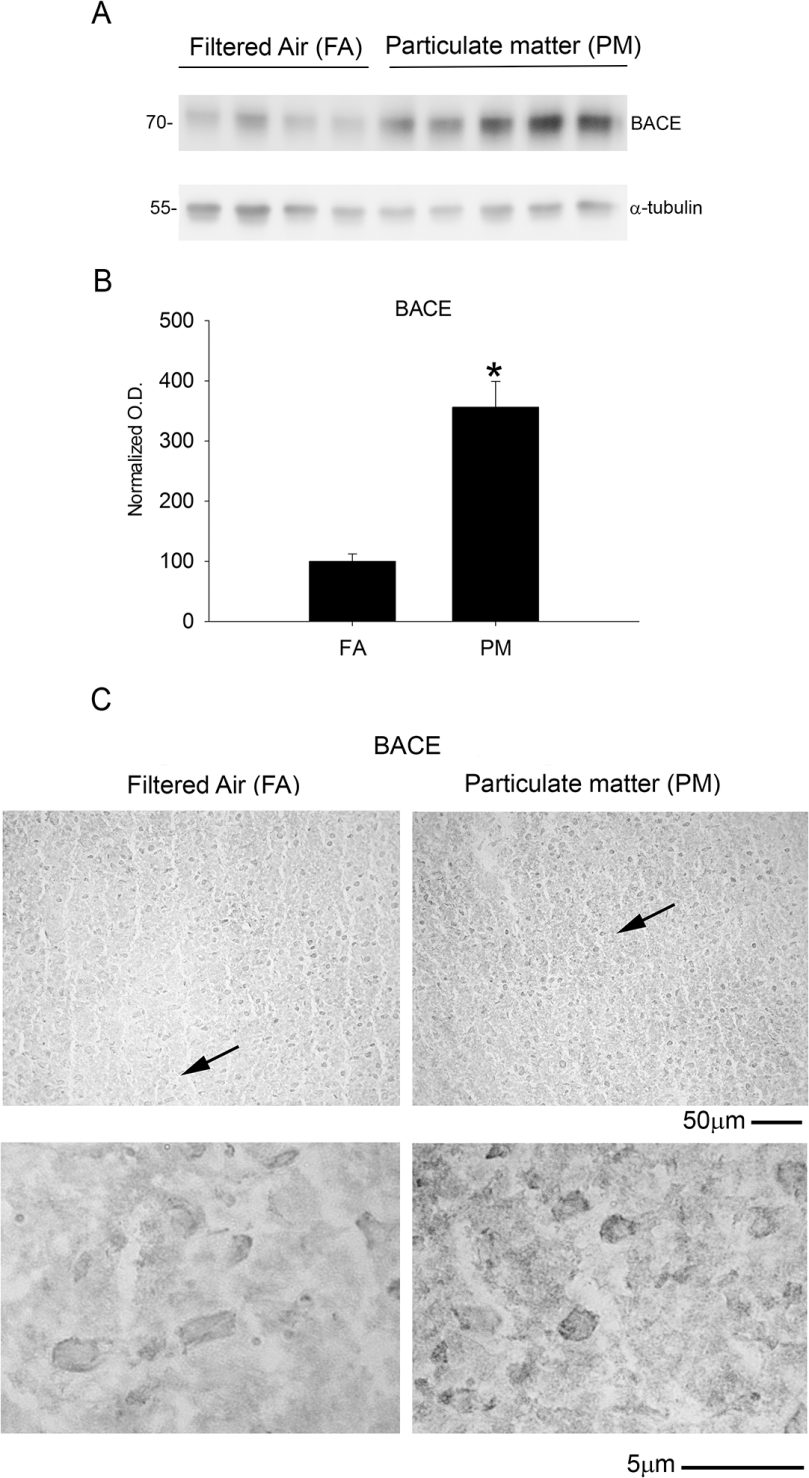
Brain beta-site APP cleaving enzyme (BACE) protein levels increased after 9 month exposure to airborne particulate matter. Brains of 9 month exposed filtered air (FA) or PM_2.5_ (PM) mice were collected with the left hemisphere fixed for immunohistochemistry and the right temporal cortices collected for biochemical analysis. *A*. Temporal cortex lysates from FA and PM brains were used for western blot analysis using anti-BACE antibody and α-tubulin (loading control). *B*. BACE optical densities were normalized to their respective loading controls, averaged (+/-SD), and graphed, *p<0.05. *C*. Brains were immunostained using anti-BACE antibody. Images shown are representative from the temporal cortex. Arrows indicate regions taken for higher magnification.

To further determine the mechanism(s) underlying the elevation of Aβ levels, we quantified protein levels of the amyloid precursor protein (APP) and one of the cleavage enzymes responsible for Aβ formation, beta-site APP cleaving enzyme (BACE). The 9 month PM_2.5_ exposure significantly reduced temporal cortex APP protein levels (Fig 3) and immunoreactivity compared to controls (Fig 3). These results were supported by a parallel increase in temporal cortex BACE protein levels and immunoreactivity with 9 month PM_2.5_ exposure (Fig 4). Interestingly, none of these changes were significant with 3 month PM_2.5_ exposure (S1 Fig). Collectively, these data demonstrate that an increase in protein levels of the APP protease, BACE, observed with 9 month airborne particulate matter exposure correlated with decreased levels of full length APP and increased proteolytic fragment, Aβ 1–40.

### Exposure to airborne particulate matter increased protein levels of the post-synaptic marker, PSD-95

**Fig 5:**
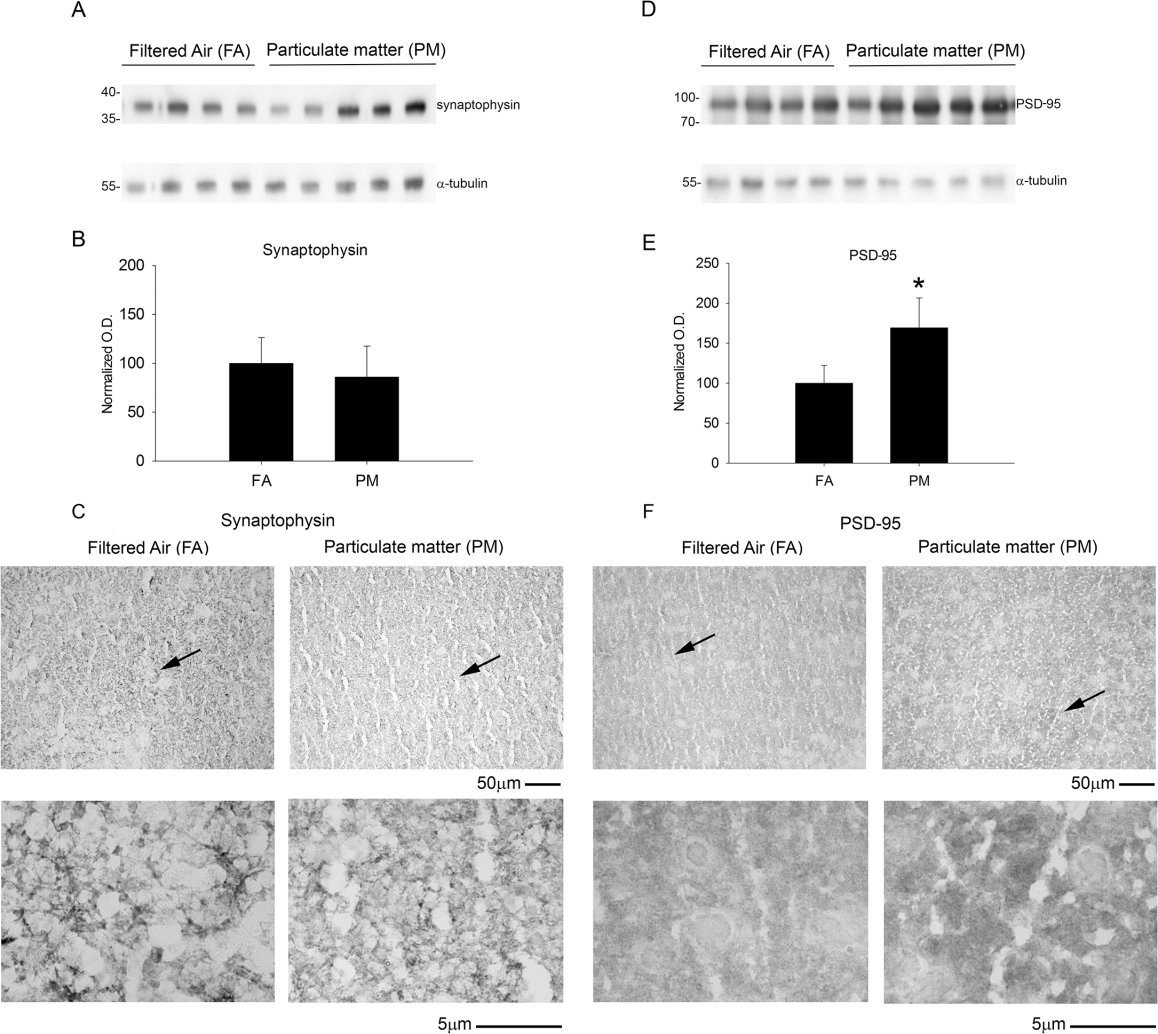
Brain post-synaptic marker, PSD-95, protein levels increased with 9 month airborne particulate matter exposure with no change in the pre-synaptic protein marker, synaptophysin. Brains of 9 month exposed filtered air (FA) or PM_2.5_ (PM) mice were collected with the left hemisphere fixed for immunohistochemistry and the right temporal cortices collected for biochemical analysis. Temporal cortex lysates from FA and PM brains were used for western blot analysis using *A*, anti-synaptophysin, *D*, anti-PSD-95 and α-tubulin (loading control) antibodies. Optical densities for *B*, synaptophysin and *E*, PSD-95 were normalized to their respective loading controls, averaged (+/-SD), and graphed, *p<0.05. Brains were immunostained using *C*, anti-synaptophysin and *F*, anti-PSD-95 antibodies. Images shown are representative from the temporal cortex. Arrows indicate regions taken for higher magnification.

To determine whether significantly elevated Aβ levels correlated with substantial synaptic changes, we quantified protein levels of pre- and post-synaptic markers using western blot analysis. There was no significant difference in the pre-synaptic marker, synaptophysin, with 9 month PM_2.5_ exposure (Fig 5). However, a significant elevation of the post-synaptic marker, PSD-95, was observed by both western blot and immunostaining after 9 month PM_2.5_ exposure (Fig 5). These observations demonstrate that 9 month exposure to PM_2.5_ had a selective effect on the post-synaptic and not pre-synaptic markers.

### Exposure to airborne particulate matter modestly increased cytokine levels but did not alter glial or vascular protein markers of activation

**Fig 6:**
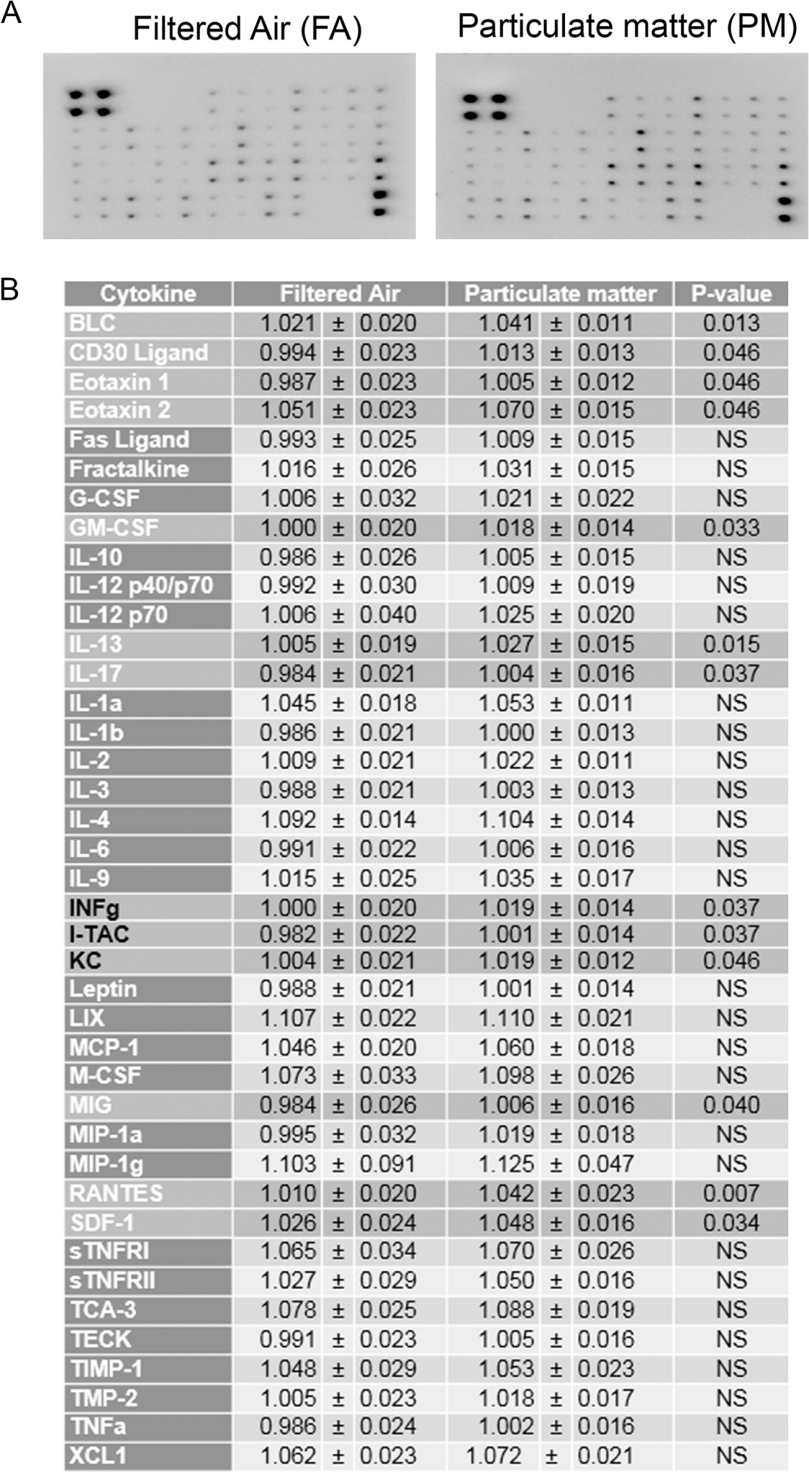
Brain inflammatory cytokines were modestly increased with 9 month airborne particulate matter exposure. Brains of 9 month exposed filtered air (FA) or PM_2.5_ (PM) mice were collected with the left hemisphere fixed for immunohistochemistry and the right temporal cortices collected for biochemical analysis. *A*. Temporal cortex lysates from FA and PM brains were used for antibody-based commercial cytokine array analysis. A representative array is shown. *B*. The optical densities for each cytokine were averaged (+/-SD).

**Fig 7:**
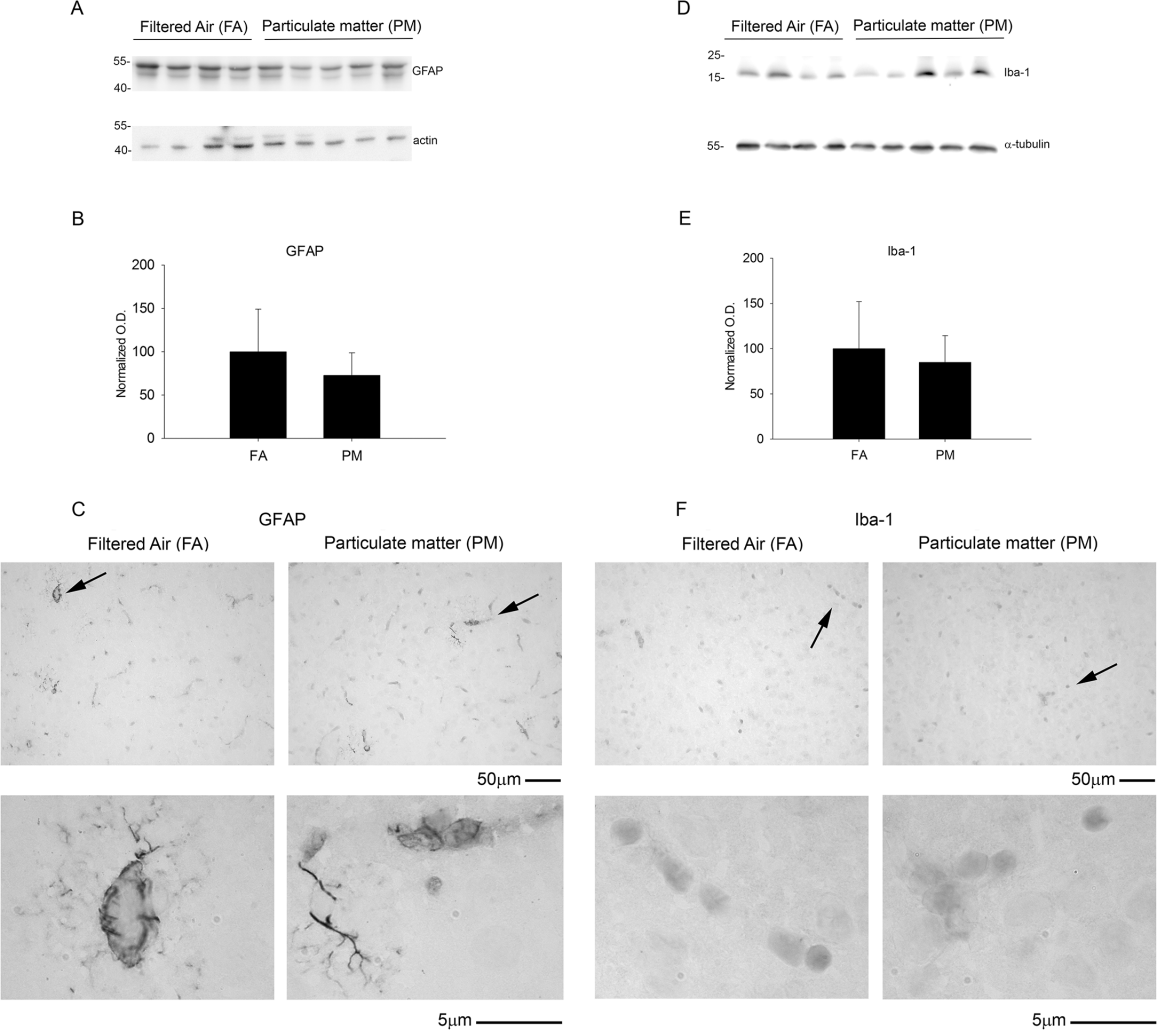
Brain astrocytic (GFAP) and microglial (Iba-1) protein markers were unaltered with 9 month airborne particulate matter exposure. Brains of 9 month exposed filtered air (FA) or PM_2.5_ (PM) mice were collected with the left hemisphere fixed for immunohistochemistry and the right temporal cortices collected for biochemical analysis. Temporal cortex lysates from FA and PM brains were used for western blot analysis using *A*, anti-GFAP, *D*, anti-Iba-1 and α-tubulin (loading control) antibodies. Optical densities for *B*, GFAP and *E*, Iba-1 were normalized to their respective loading controls, averaged (+/-SD), and graphed. Brains were immunostained using *C*, anti-GFAP and *F*, anti-Iba-1 antibodies. Images shown are representative from the temporal cortex. Arrows indicate regions taken for higher magnification.

**Fig 8:**
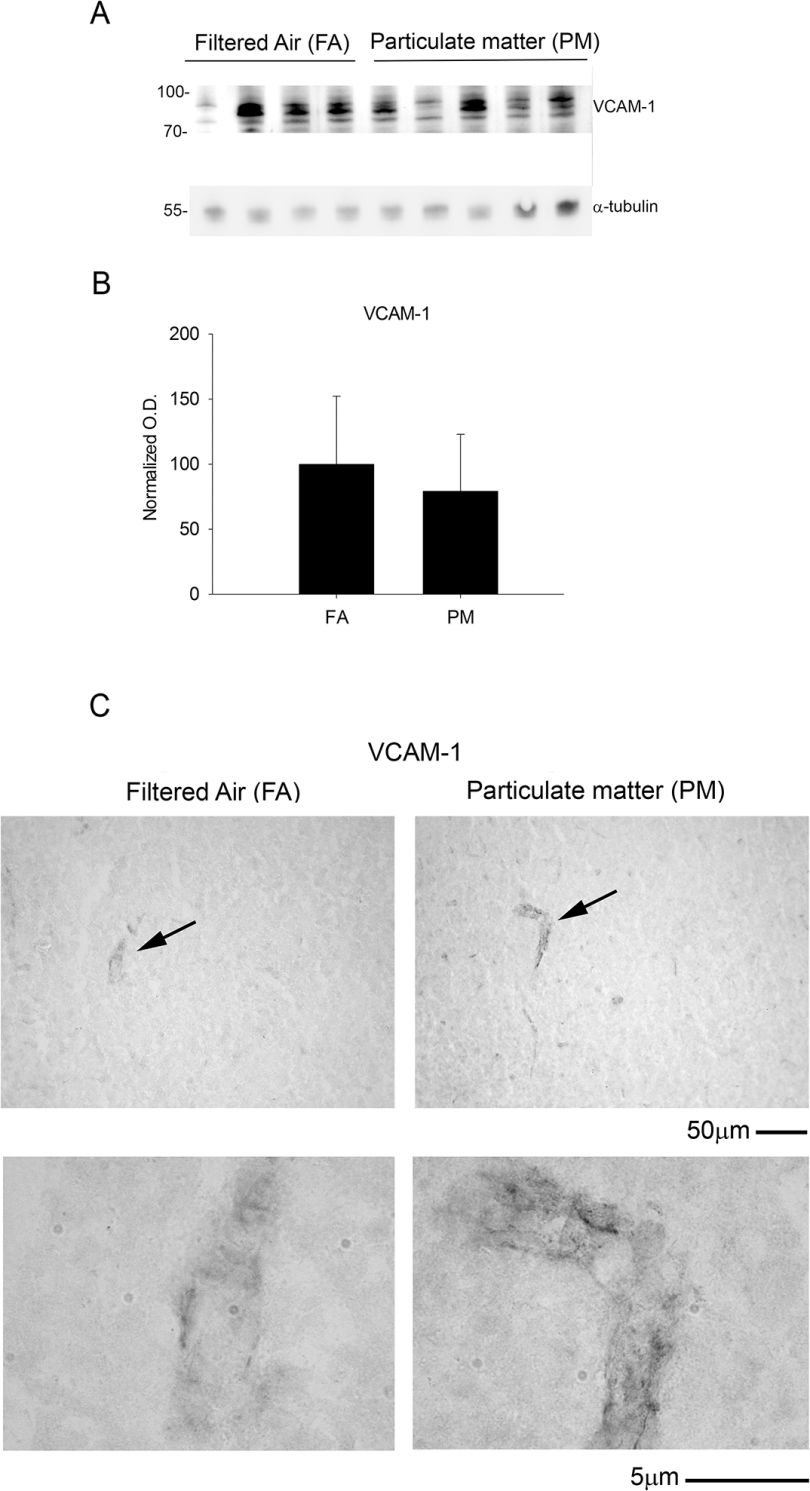
Vascular protein marker, VCAM-1, was unaltered with 9 month airborne particulate matter exposure. Brains of 9 month exposed filtered air (FA) or PM_2.5_ (PM) mice were collected with the left hemisphere fixed for immunohistochemistry and the right temporal cortices collected for biochemical analysis. *A*. Temporal cortex lysates from FA and PM brains were used for western blot analysis using anti-VCAM antibody and α-tubulin (loading control). *B*. VCAM optical densities were normalized to their respective loading controls, averaged (+/-SD), and graphed. *C*. Brains were immunostained using anti-VCAM antibody. Images shown are representative from the temporal cortex. Arrows indicate regions taken for higher magnification.

To determine if enhanced APP processing and increased PSD-95 levels correlated with an increase in chronic brain inflammatory response, we performed a cytokine profile analysis and quantified protein markers of glial and vascular activation. Using a mouse inflammatory cytokine array, we profiled 40 inflammatory cytokines and found a modest increase in a subset of cytokines that largely belong to the chemokine-chemoattractant class (Fig 6). On the other hand, we did not observe any significant change in GFAP and Iba-1 levels, markers for astrocytic and microglial activation, respectively (Fig 7). Similarly, no significant differences were observed in the vascular activation marker, VCAM-1, between 9 month PM_2.5_ exposure and controls (Fig 8). These data suggest that PM_2.5_ induced a mild chemotactic response in the brain. However, no changes in glial markers of activation were observed by 9 months of exposure.

### Exposure to airborne particulate matter increased cyclooxygenase-1 and -2 protein levels but did not alter markers of oxidative or nitrosative stress or DNA methylation

**Fig 9:**
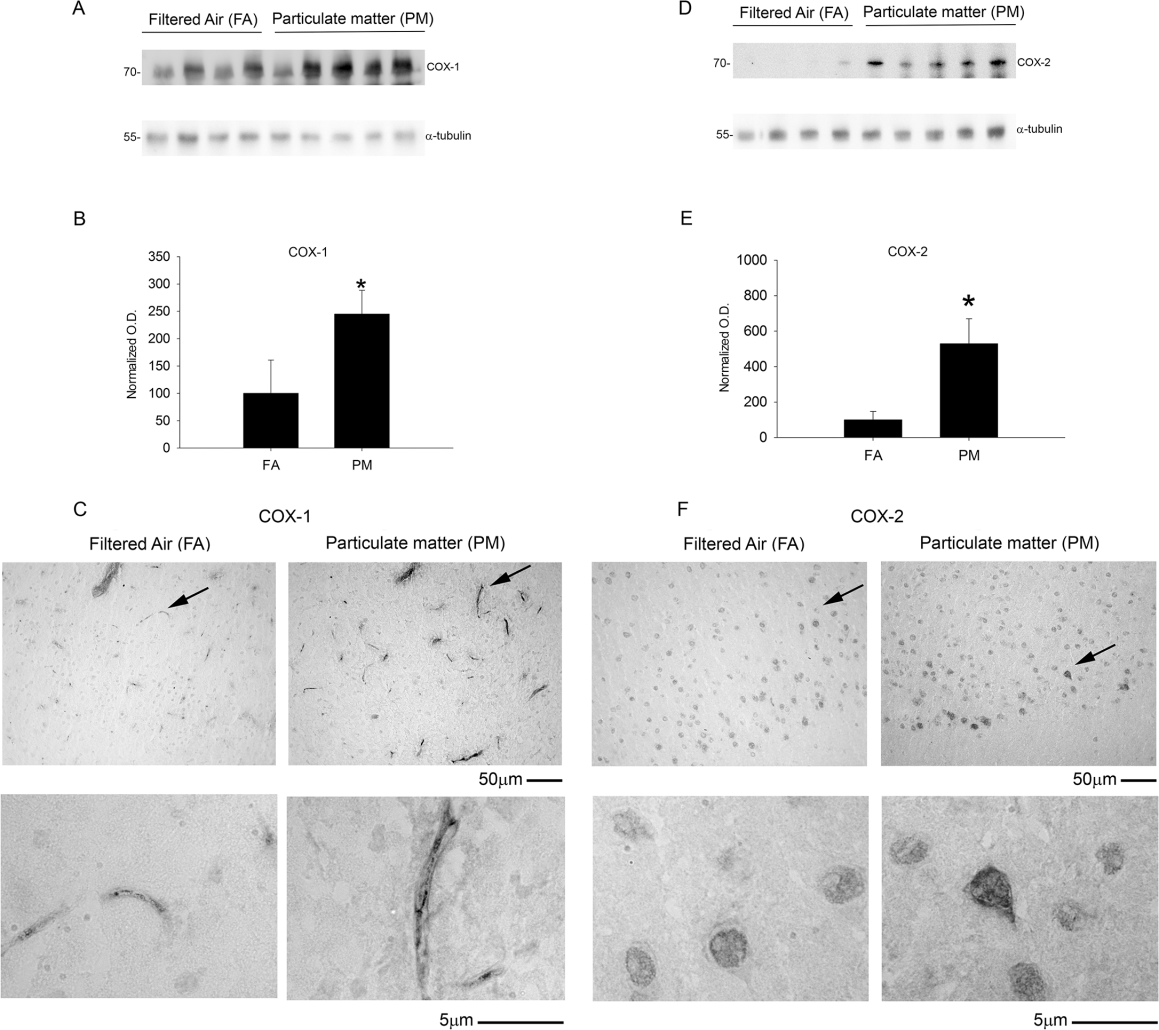
Brain cyclooxygenase enzyme (COX-1 and COX-2) protein levels increased with 9 month airborne particulate matter exposure. Brains of 9 month exposed filtered air (FA) or PM_2.5_ (PM) mice were collected with the left hemisphere fixed for immunohistochemistry and the right temporal cortices collected for biochemical analysis. Temporal cortex lysates from FA and PM brains were used for western blot analysis using *A*, anti-COX-1, *D*, anti-COX-2 and α-tubulin (loading control) antibodies. Optical densities for *B*, COX-1 and *E*, COX-2 were normalized to their respective loading controls, averaged (+/-SD), and graphed, *p<0.05. Brains were immunostained using *C*, anti-COX-1 and *F*, anti-COX-2 antibodies. Images shown are representative from the temporal cortex. Arrows indicate regions taken for higher magnification.

**Fig 10:**
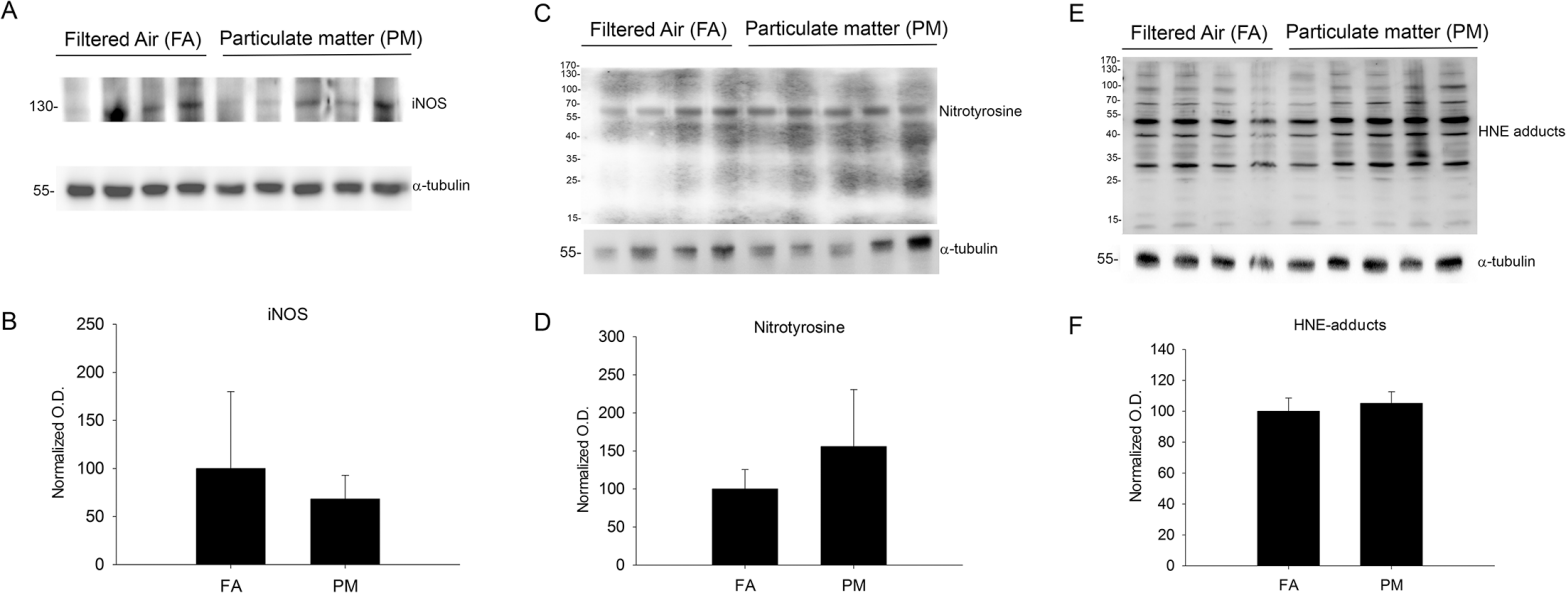
Brain oxidative and nitrosative stress protein marker levels were not altered with 9 month airborne particulate matter exposure. Brains of 9 month exposed filtered air (FA) or PM_2.5_ (PM) mice were collected with the left hemisphere fixed for immunohistochemistry and the right temporal cortices collected for biochemical analysis. Temporal cortex lysates from FA and PM brains were used for western blot analysis using *A*, anti-inducible nitric oxide synthase (iNOS), *C*, nitrated tyrosines (nitrotyrosine), *E*, 4-hydroxynonenal protein Michael adducts (HNE adducts) and α-tubulin (loading control) antibodies. Optical densities for *B*, iNOS and *E*, nitrotyrosine, and *F*, HNE adducts were normalized to their respective loading controls, averaged (+/-SD), and graphed.

**Fig 11:**
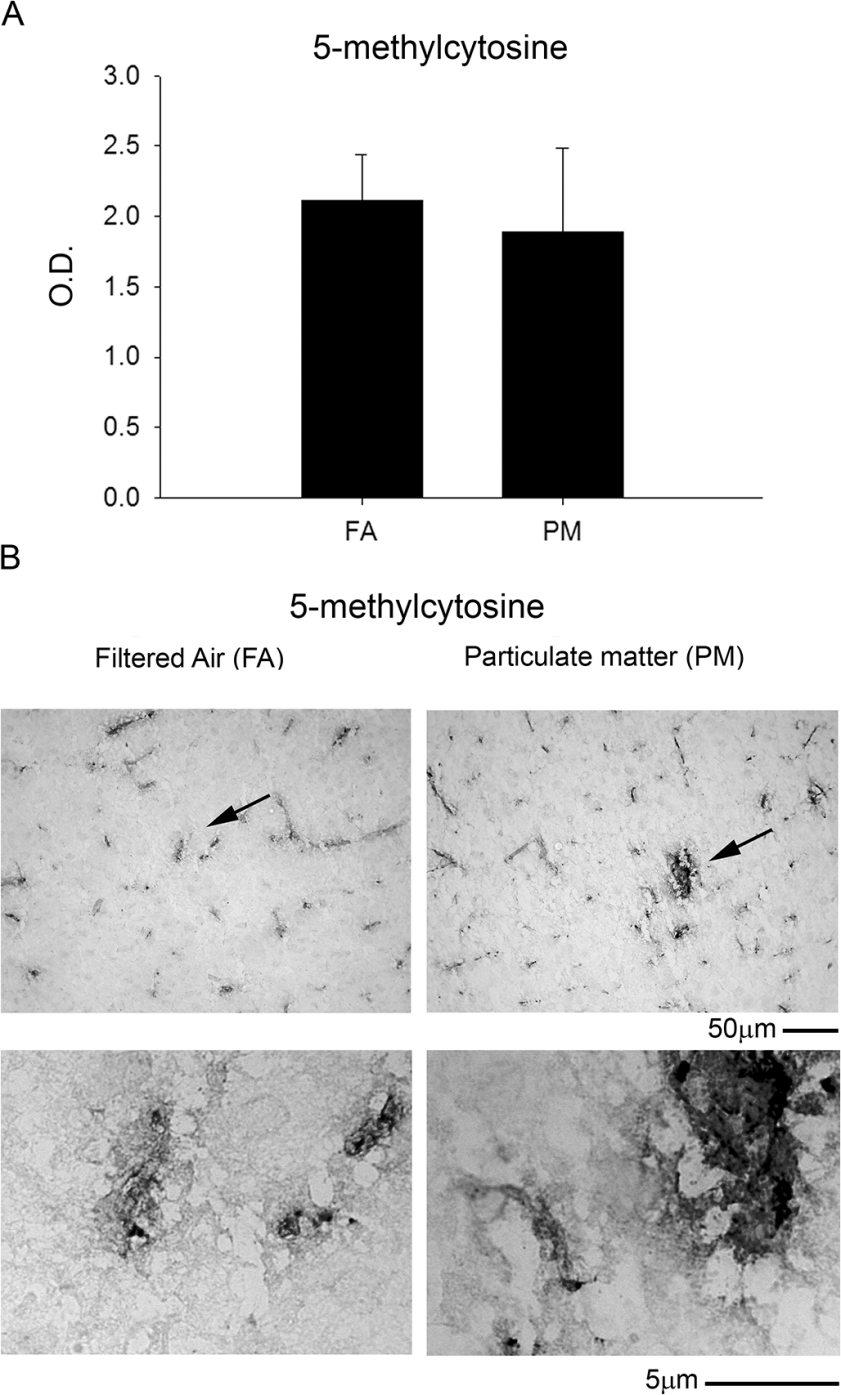
Brain DNA methylation was not altered with 9 month airborne particulate matter exposure. Brains of 9 month exposed filtered air (FA) or PM_2.5_ (PM) mice were collected and the right parietal cortices were collected for DNA isolation. *A*. A 5-methylcytosine DNA ELISA was performed from isolated DNA (100ng/brain) from FA and PM brains. *B*. Optical densities were averaged (+/-SD) and graphed. *C*. Brains were immunostained using anti-5 methyl-cytosine antibody. Images shown are representative from the temporal cortex. Arrows indicate regions taken for higher magnification.

We next examined alternative proinflammatory and stress pathways that might be influenced by PM_2.5_ exposure including the prostaglandin forming cyclooxygenase enzymes (COX-1/2), inducible nitric oxide synthase enzyme (iNOS), a nitrosative protein stress marker (nitrotyrosine) and an oxidative protein stress marker (HNE-adducts). We observed significant increases in both COX-1 and COX-2 protein levels by western blot as well as increased immunostaining in the temporal cortex with 9 month PM_2.5_ exposure (Fig 9). This was not observed in the 3 month PM_2.5_ exposure mice (S2 Fig). None of the other stress markers, iNOS, nitrotyrosine, or HNE-adducts, were significantly altered with 9 month PM_2.5_ exposure (Fig 10). Based upon increasing evidence that DNA methylation changes are present after particulate matter exposure [14–15], we next quantified 5-methylcytosine levels via ELISA from DNA isolated from parietal cortices. No significant differences were observed between FA and PM_2.5_ exposed mouse brains (Fig 11). These data demonstrated that a modest increase in the chemotactic cytokine profile and Aβ levels in 9 month PM2.5 exposure brains correlated with increased levels of the prostaglandin producing enzymes, COX-1 and COX-2.

## Discussion

In this pilot study we demonstrated that long-term exposure to ambient airborne particulate matter modestly increased cytokine, Aβ, and COX-1/2 levels associated with early AD-like pathology in rodent brain. Of the two hallmark features of the AD brain examined, chronic inhalation of airborne particulate matter promoted specifically the amyloid-associated pathology. We found that 9 months of exposure to particulate matter increased BACE levels and decreased APP levels correlating with a significant increase in Aβ 1–40. Since an increase in BACE protein levels and decrease in APP protein levels does not necessarily indicate increased APP processing, future work will need to quantify changes in BACE activity as well as Aβ brain clearance to better understand the observed increase in Aβ 1–40 levels. These changes correlated with increases in COX-1, COX-2, and PSD-95 protein levels and a modest increase in the chemotactic cytokine profile. However, similar changes were absent in the brains of mice exposed to ambient particulate matter inhalation for only 3 months. These data support the hypothesis that prolonged exposure to NAAQS levels of ambient airborne particulate matter alters the brain inflammatory profile and promotes early AD changes.

Several observational studies in humans [9–16–20] and dogs [16–21–22] that were exposed to high levels of coarse or fine particulate matter through inhalation of polluted air, strongly implicate the role of air pollution and inflammatory changes in the development of AD pathology. However, controlled interventional studies looking at the influence of long-term exposure to ambient levels of particulate matter on the brain inflammatory response and AD pathology are lacking. The OASIS-1 system used in this study is a mobile, versatile aerosol concentrator and enrichment system that allows investigation of the health effects of environmental exposure to air-pollution in a controlled and physiologically relevant manner. In this study, we demonstrated early AD-like pathology with 9 but not 3 months of inhalation of particulate matter. Moreover, we used wild type C57BL/6 mice in this study which are not genetically modified for disease predisposition, further underscoring the pathogenic potential of chronic exposure to NAAQS levels of PM_2.5_. A controlled system, such as ours, used with more aggressive transgenic mouse models of AD will allow for association studies between specific air pollutants and measurable outcomes of AD pathology and cognitive decline.

Although acute brain inflammation may be considered a general protective mechanism, chronic inflammation has been considered an important component contributing to progression of AD [4–23]. Exposure to PM_2.5_ and other air pollutants has the potential to produce systemic immune dysfunction. Infiltration of immune mediators or PM_2.5_ itself through either the circulation or the olfactory nerve to the brain can result in activation of brain inflammatory mediators and gliosis [7–24]. Indeed, others have shown that mice exposed to concentrated PM_2.5_ for two weeks show elevated IL-1α and TNF-α levels, and NF-κB activation [25]. In this study, we demonstrated that NAAQS PM_2.5_ levels present in ambient air caused a modest elevation in brain inflammation. The minimal changes we observed may be explained by the low levels of PM_2.5_ concentration used in this study and we hypothesize that longer exposure or more concentrated exposure would produce exacerbations of the changes we observed. In addition, we appreciate that some of the cytokine changes observed may have been contributed by lingering blood in the tissue based upon our lack of perfusion prior to collection. In addition, we focused our efforts on temporal cortex based upon its relevance for AD-like pathology changes in humans and transgenic mouse models of disease. We recognize that this may not be the most relevant brain region to examine for early inflammatory changes in the brain due to particulate matter inhalation. Although this pilot study provided promising and significant changes, future work with larger animal numbers, behavioral analysis, additional time point collections, and multi-brain region analysis at each time point is needed to better define the contribution of particulate matter inhalation in our model to AD-like changes. Moreover, future effort in AD mouse models will also need to be done to determine the precise mechanism(s) of how varying levels and exposure periods of PM_2.5_ inhalation may result in central nervous system inflammation across different brain regions and exacerbation of AD pathology.

Despite modest changes in the inflammatory cytokines and no differences in oxidative and nitrosative stress markers, significant increases of COX-1 and COX-2 protein levels were observed in this study. AD patients also have reportedly increased COX-2 levels in hippocampal neurons [26]. In addition, human subjects [9] and dogs [10] from areas with high PM_2.5_ levels have been characterized with elevated COX-2 mRNA levels as a robust response. Collectively, data from both our study and others implicates key roles for COX-1 and COX-2 proteins in the development of PM-induced AD pathology perhaps serving as an early or initiating change. In this regard, the beneficial role of nonsteroidal anti-inflammatory drugs (NSAIDs) in AD has been debatable. However, epidemiologic data does support the idea that prolonged NSAID use very early in disease reduces risk [4] further corroborating the current findings. It is even feasible that the PM_2.5_-mediated increase in risk of AD has a unique component of early COX-1/2 activity that might distinguish it from other causes/risks of disease. One exciting opportunity in future work will be to quantify specific prostaglandin levels across brain regions and exposure periods to correlate with the protein changes in not only COX-1/2 but also the AD-like pathology. This may support the hypothesis that prostaglandins have a role in regulating the AD-like changes and stimulate further effort in COX-1/2 inhibitory or prostaglandin receptor antagonist prevention studies.

Based upon the increasing evidence that DNA methylation changes occur in AD brains and in response to particulate matter inhalation, we expected to see significant change in 5-methylcytosine DNA levels to correlate with the various protein changes we observed. However, it is likely that the changes in global DNA 5-methylcytosine levels we examined in our general ELISA assessment strategy was simply not sensitive enough for detecting changes in specific gene methylation patterns. Environmental factors are known to induce several epigenetic modifications such as DNA methylation and acetylation or methylation of histone proteins [27]. Of these, the most well characterized epigenetic modification is DNA methylation. This regulates gene silencing and mounting evidence suggests strong correlation between environmental exposure to air pollutants and altered DNA methylation [14–15]. Further, DNA methylation mediated changes in gene expression are associated with normal aging and several human diseases including AD [28]. While global DNA hypermethylation is observed in AD patients, promoter regions of the COX-2 gene are hypomethylated suggestive of increased expression [29]. Moreover, both BACE and PS1 genes are involved in APP processing and are regulated by DNA methylation. Depletion of the universal methyl donor S-adenosyl methionine from human neuronal cultures results in increased PS1 and BACE levels with an increase in Aβ production [30]. Future work will require gene selective assessment of both hypomethylation and hypermethylation patterns to determine whether this correlates with a change in mRNA and expression of any of the protein changes we observed.

In conclusion, we demonstrate that prolonged exposure to NAAQS levels of airborne particulate matter alters brain cytokine levels and promotes development of early AD-like pathology and increased COX-1/2 protein levels. This study provides a reliable model system to replicate ambient particulate matter exposure in a controlled manner. Information gleaned by combining this exposure system with animal models of AD may be helpful in developing preventive and targeted therapeutic strategies against, particularly, environmental risk factors of disease.

## Supporting Information

S1 Fig Brain APP and BACE protein levels were not altered with 3 month airborne particulate matter exposure. Brains of 3 month exposed filtered air (FA) or PM_2.5_ (PM) mice were collected with the left hemisphere fixed for immunohistochemistry and the right temporal cortices collected for biochemical analysis. Temporal cortex lysates from FA and PM brains were used for western blot analysis using *A*, anti-APP (Y188), BACE, and α-tubulin (loading control) antibodies. Optical densities for *B*, APP and *C*, BACE were normalized to their respective loading controls, averaged (+/-SD), and graphed.
(TIF)

S2 Fig Brain cyclooxygenase enzyme (COX-1 and COX-2) and post-synaptic marker, PSD-95, protein levels were not altered with 3 month airborne particulate matter exposure. Brains of 3 month exposed filtered air (FA) or PM_2.5_ (PM) mice were collected with the left hemisphere fixed for immunohistochemistry and the right temporal cortices collected for biochemical analysis. Temporal cortex lysates from FA and PM brains were used for western blot analysis using *A*, anti-COX-1, COX-2, PSD-95, and α-tubulin (loading control) antibodies. Optical densities for *B*, COX-1 and *B*, COX-2, and *C*, PSD-95 were normalized to their respective loading controls, averaged (+/-SD), and graphed.
(TIF)
