# Supplementary figures and images for: A Pilot Study to Assess Effects of Long-Term Inhalation of Airborne Particulate Matter on Early Alzheimer-Like Changes in the Mouse Brain
Source: PLoS One. 2015 May 20;10(5):e0127102. doi: 10.1371/journal.pone.0127102 (PMC4439054; doi:10.1371/journal.pone.0127102)

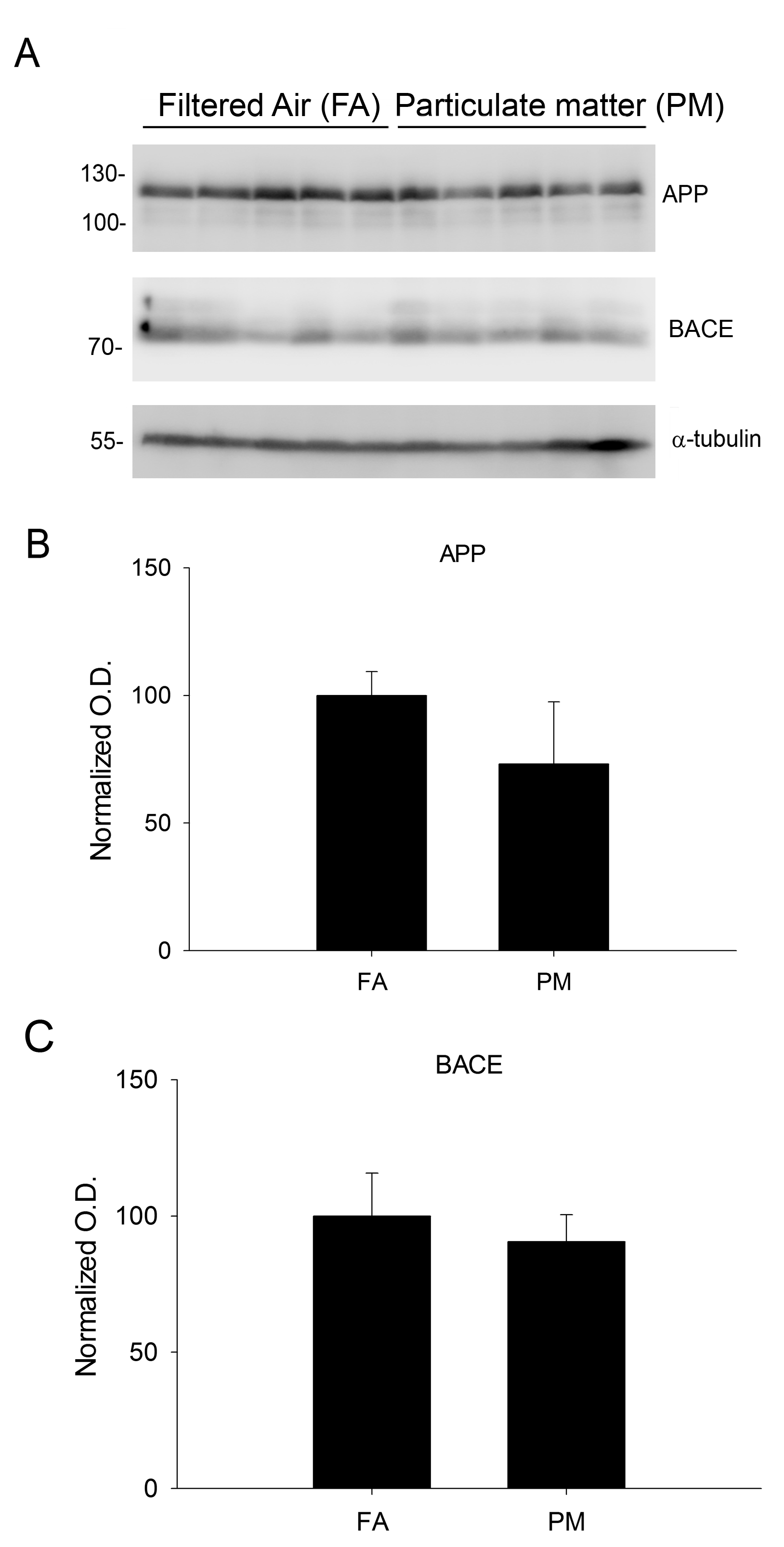

Supplement: S1 Fig — Brains of 3 month exposed filtered air (FA) or PM2.5 (PM) mice were collected with the left hemisphere fixed for immunohistochemistry and the right temporal cortices collected for biochemical analysis. Temporal cortex lysates from FA and PM brains were used for western blot analysis using A, anti-APP (Y188), BACE, and α-tubulin (loading control) antibodies. Optical densities for B, APP and C, BACE were normalized to their respective loading controls, averaged (+/-SD), and graphed. (TIF) [file pone.0127102.s001.tif]

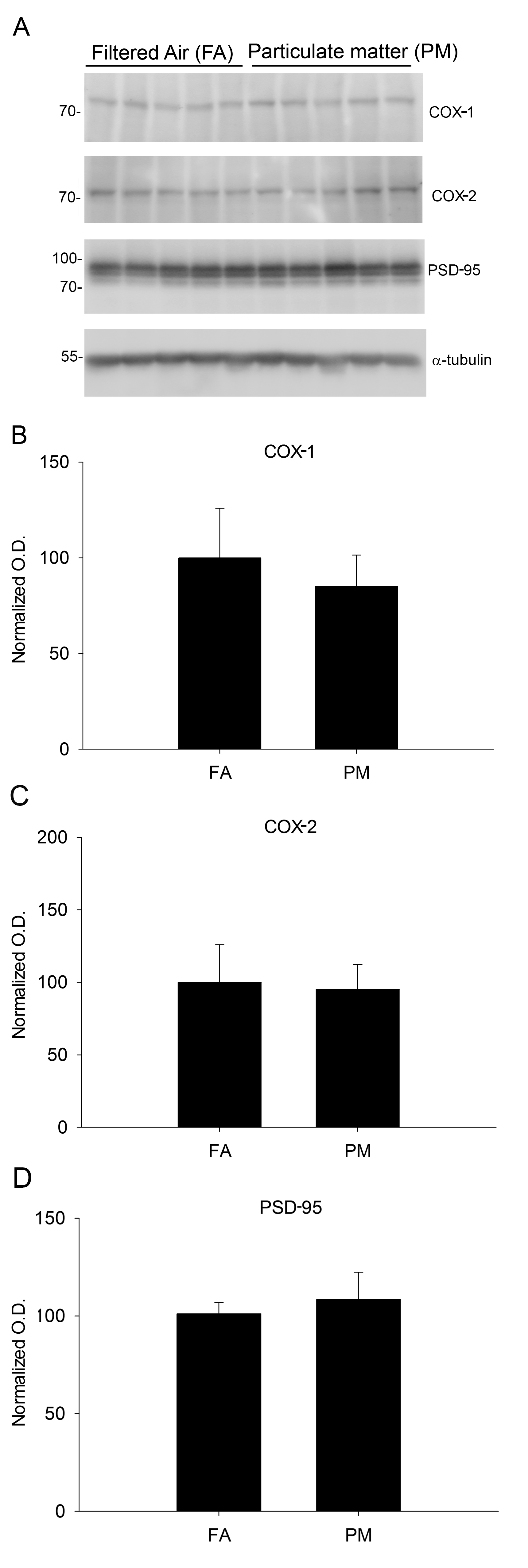

Supplement: S2 Fig — Brains of 3 month exposed filtered air (FA) or PM2.5 (PM) mice were collected with the left hemisphere fixed for immunohistochemistry and the right temporal cortices collected for biochemical analysis. Temporal cortex lysates from FA and PM brains were used for western blot analysis using A, anti-COX-1, COX-2, PSD-95, and α-tubulin (loading control) antibodies. Optical densities for B, COX-1 and B, COX-2, and C, PSD-95 were normalized to their respective loading controls, averaged (+/-SD), and graphed. (TIF) [file pone.0127102.s002.tif]
